# DNA Damage, Neurodegeneration, and Synaptic Plasticity

**DOI:** 10.1155/2016/1206840

**Published:** 2016-05-25

**Authors:** Daniela Merlo, Inmaculada Cuchillo-Ibañez, Rosanna Parlato, Gerhard Rammes

**Affiliations:** ^1^Department of Cell Biology and Neuroscience, Istituto Superiore di Sanità, Viale Regina Elena 299, 00161 Rome, Italy; ^2^Instituto de Neurociencias, CSIC-UMH, Avenida Ramón y Cajal s/n, 03550 Sant Joan d'Alacant, Spain; ^3^Institute of Applied Physiology, Ulm University, 89081 Ulm, Germany; ^4^Institute of Anatomy and Medical Cell Biology, Heidelberg University, 69120 Heidelberg, Germany; ^5^Klinikum Rechts der Isar, Department of Anesthesiology, Technische Universität München, Ismaninger Strasse 22, 81675 Munich, Germany

This special issue includes four reviews and four original research articles focusing on the physiological and pathological role of DNA damage and repair mechanisms, synaptic plasticity, and neurodegeneration. Moreover, the emerging roles played in memory and neurodegeneration by different factors involved in the stress response, chromatin remodeling, and nucleolar function are specifically addressed. Finally, the present collection includes articles outlining current treatments for neurodegenerative diseases including Alzheimer's disease (AD) and Niemann-Pick type C disease (NPC).

Unrepaired DNA lesions and deficiencies in DNA repair systems are implicated in the progressive neuronal loss observed in many neurodegenerative pathologies although neuronal DNA damage and repair may also play a role in cognitive function and dysfunction. The formation of double strand breaks (DSBs), the most lethal form of DNA damage, may be a physiological process that modifies chromatin organization and gene expression involved in information processing, learning, and memory and may be caused by normal brain activity. Conversely, pathologically elevated *β*-amyloid peptide (A*β*), a major culprit for the pathogenesis of AD, worsens DNA damage by eliciting aberrant synaptic activity.

We still have limited understanding of the precise molecular mechanisms underlying failures in memory formation and consolidation and the synaptic dysfunction associated with the early onset of neurodegenerative diseases. Therefore, this special issue aims to contribute to the knowledge on these mechanisms involved in both cognitive impairment and neurodegeneration to indicate novel therapeutic targets.

In the review article by L. Narciso et al., “The Response to Oxidative DNA Damage in Neurons: Mechanisms and Disease,” the authors outline the notion that the mechanisms controlling genome stability are of key importance in the development and function of the nervous system. They discuss and present different neurological diseases characterized by impairment of DNA repair pathways focusing on base excision repair (BER). Moreover, they stress the evidence that the mechanisms controlling DNA damage in neurons may vary depending on the brain region and suggest the need for further investigation to determine whether differences in the response to DNA damage underlie the brain-region selectivity observed in neurodegenerative diseases.

Factors known to function in DNA recombination/repair machineries have been shown to play a role in long-term memory (LTM). The research article by E. Castro-Pérez et al., “Identification and Characterization of the V(D)J Recombination Activating Gene 1 in Long-Term Memory of Context Fear Conditioning,” reports that the* V(D)J* recombination activating gene 1 (RAG1), which encodes a factor that introduces DSBs in immunoglobulin and T-cell receptor genes, is induced in the amygdala, but not in the hippocampus, after context fear conditioning. In functional studies, ablation of RAG1 expression causes a significant impairment in LTM, thus suggesting that RAG1 may play a role in LTM consolidation. This work further supports the notion that DNA recombination/repair machineries might be involved in learning and memory processes. Interestingly, the authors suggest that an integrated control of the introduction of DSBs, DNA repair, DNA rearrangement, epigenetics, and transcriptional and translational mechanisms may orchestrate gene regulation in memory formation.

A*β* is the key player in the amyloid-cascade hypothesis of AD which appears to be associated with DSB induction in aging and AD. The article by E. Gruz-Gibelli et al., “The Vitamin A Derivative All-Trans Retinoic Acid Repairs Amyloid-*β*-Induced Double-Strand Breaks in Neural Cells and in the Murine Neocortex,” indicates a role of all-trans retinoic acid (RA), a derivative of vitamin A, in the repair of A*β*-induced DBSs. The authors test the DNA and the cellular protection activity of RA both on neuronal SH-SY5Y cells and on astrocytic DI TNC1 cells and, in addition, on extracts from cortex of young and old mice. The authors suggest that RA, besides increasing cell viability in the cortex of young and aged mice, might also target DNA repair of genes involved in synaptic maintenance. Therefore, exploring mechanisms involved in A*β*-induced DSBs might provide additional means to target pathological A*β*-induced changes.

Epigenetic silencing of rDNA by DNA methylation is a common feature of mild cognitive impairment (MCI) and AD and it is considered an emerging disease marker. Interestingly, rDNA silencing in the nucleolus perturbs diverse nucleolar functions including global chromatin regulation and biogenesis of ribosomes. Among the chromatin-remodeling enzymes, poly(ADP-ribose) polymerase-1 (PARP-1) may control synaptic plasticity and memory consolidation by regulating the expression of immediate early genes and rDNA transcription. Recently, it has been shown that the maintenance of the late phase of long-term potentiation (L-LTP), a model for long-term memory, requires nucleolar integrity and the expression of new rRNAs. The research article by J. Zeng et al., “Nucleolar PARP-1 Expression Is Decreased in Alzheimer's Disease: Consequences for Epigenetic Regulation of rDNA and Cognition,” shows that the nucleolar PARP-1 may be a sensitive marker of synaptic deficits in AD. The authors suggest a novel role for PARP-1 dysregulation in AD pathology by showing that PARP-1 positive nucleolar staining in CA1 and CA4 hippocampal pyramidal neurons of AD patients is significantly reduced compared to controls. They also propose an intriguing model in which the loss of nucleolar PARP-1, resulting in DNA methyltransferase activation, might account for the rDNA silencing observed in AD.

AD is a complex multifactorial disorder which may require complex approaches to treatment. Early disease detection, combination therapies, and lifestyle choices are all likely contributors to the successful eradication of the pathology. In the review article “Current Research Therapeutic Strategies for Alzheimer's Disease Treatment,” J. Folch et al. consider the effectiveness of treatments aimed at reducing A*β* production through both the inhibition of *β* and *γ* secretase enzymes and the dissolution of existing cerebral A*β* plaques to be modest. Interestingly, they present alternative strategies centered on the inhibition of the downstream A*β* signaling, particularly acting at synaptic level. The interaction of A*β* and prion protein (PrPC) activates Fyn kinase which then modifies synaptic signaling through NMDA glutamate receptors. This mechanism underlies excitotoxicity and dendritic spine loss. Thus, the authors propose Fyn kinase blockers Masitinib and Saracatinib as effective molecules in treating AD symptoms in experimental mouse models of the disease. In fact, Saracatinib is currently in Phase II and Masitinib is in Phase III clinical trials for mild-to-moderate AD.

In the research paper by G. D'Arcangelo et al., “Miglustat Reverts the Impairment of Synaptic Plasticity in a Mouse Model of NPC Disease,” the authors investigate in NPC, a rare disease with progressive neurological deterioration and cognitive decline until severe dementia, the mechanism of action of Miglustat, a recent approved drug for the treatment of the disease. In particular, they study synaptic plasticity phenomena and evaluate ERKs activation in the hippocampus of NPC1−/− mice, a well described animal model of the disease. The authors show an impairment of LTP in NPC1−/− mouse slices which is associated with lack of ERKs phosphorylation. They also find that* in vivo* Miglustat administration in NPC1−/− mice can rescue synaptic plasticity deficits, restore ERKs activation, and counteract hyperexcitability. Overall, these data indicate that Miglustat may be effective for treating the neurological deficits associated with NPC, such as seizures and dementia.

Parkinson's disease (PD) is the second most common neurodegenerative disorder. This is characterized by the progressive loss of midbrain dopaminergic (mDA) neurons in the substantia nigra pars compacta (SNpc) and the presence of *α*-synuclein-containing protein aggregates termed Lewy bodies (and/or Lewy neurites) in affected neurons. Several transcription factors, playing a role in development and survival of mDA neurons, might be involved in the progressive loss of these neurons. The review article “Neuroprotective Transcription Factors in Animal Models of Parkinson Disease” by F.-X. B. de Thé et al. reports mechanisms by which these transcription factors control neuronal survival and activity, including genomic stability and synaptic maintenance. The authors suggest that a better understanding of these modes of action could help to identify novel neuroprotective approaches, for example, based on direct protein delivery strategies.

In the review article “Chronic Stress and Glucocorticoids: From Neuronal Plasticity to Neurodegeneration,” S. Vyas and collaborators discuss the cause-effect relationships between prolonged stress, elevated levels of glucocorticoids (GCs), and cognitive/mood related disorders including AD and PD. Particularly, the authors present a comprehensive view on the cellular mechanisms through which stress and GCs may influence the pathogenesis of AD and PD. 

We believe that this special issue, by focusing on DNA damage/repair mechanisms involved in learning and memory and neurodegeneration, will be instrumental to identify new potential approaches to design effective therapeutic strategies.

*Daniela Merlo* *Daniela Merlo* *Inmaculada Cuchillo-Ibañez* *Inmaculada Cuchillo-Ibañez* *Rosanna Parlato* *Rosanna Parlato* *Gerhard Rammes* *Gerhard Rammes*

